# A Randomized, Investigator‐Blinded, Split‐Face, Controlled Trial Assessing Efficacy and Satisfaction of CE Ferulic Serum Following Nonablative Fractional Fraxel Laser Treatment for Photoaging Skin in Chinese Population

**DOI:** 10.1111/jocd.70251

**Published:** 2025-05-25

**Authors:** Xiaolei Qin, Jinlong Zhai, Chenxi Zhou, Yanmei Wang, Mengjiao Chen, Lin Zhu, Qi Shi, Weiliang Chen, Liyuan Zhang, Xiao Luo, Kai Li

**Affiliations:** ^1^ Deyue Clinic Shenzhen China; ^2^ Dexi Cosmetic Clinic Deyi Skin Institution Xi'an China

**Keywords:** CE Ferulic serum, integrated skincare, nonablative fractional Fraxel laser

## Abstract

**Background:**

Following Nonablative fractional Fraxel laser (NAFL), a well‐established treatment for skin rejuvenation, immediate post‐procedural care is essential to address any potential complications and accelerate the healing process.

**Aims:**

To evaluate the efficacy and patient satisfaction of a topical skincare regimen containing vitamin C, vitamin E, and ferulic acid—CE Ferulic serum (CEF) following NAFL treatment in the Chinese population.

**Methods:**

In this randomized, investigator‐blinded, split‐face, controlled trial, patients with mild‐to‐moderate facial photodamage were randomized to receive CEF treatment on one side of the face and normal saline (NS) on the other side immediately after the NAFL procedure and daily during the 7‐day follow‐up. The primary endpoint was the change from baseline in erythema score on Day 7, with key secondary endpoints including changes from baseline in erythema index (EI), melanin index (MI), transepidermal water loss, skin hydration, skin sebum content, scabbing, edema, overall patient satisfaction, and post‐procedure pain.

**Results:**

In total, 50 patients (female 45/50) were enrolled in this study, with a mean age of 31.6 years. The mean change from baseline in erythema score was significantly lower on the CEF side than on the NS side on Day 7 post‐NAFL treatment (0.04 ± 0.40 vs. 0.18 ± 0.48, *p* = 0.011). The CEF side also exhibited improved changes in EI, MI, and skin hydration, as well as higher overall satisfaction and less pain compared with the NS side.

**Conclusions:**

Applying CEF after NAFL treatment reduced erythema progression, maintained skin hydration, and promoted the healing process compared with NS.

**Trial Registration:**

Chinese Clinical Trial Registry: ChiCTR2300069246

## 1 Introduction

Skin photoaging, a premature skin aging process caused by ultraviolet (UV) irradiation, is characterized by several skin concerns, such as hyperpigmentation, wrinkling, loss of skin elasticity, or skin dryness [1]. Laser resurfacing technology is a common and effective method to achieve skin rejuvenation through a focal photothermal action, restoring youthful skin [2, 3]. The lasers create microthermal zones in the skin and stimulate the repair process to promote the synthesis of collagen, thereby achieving the purpose of skin tissue remodeling [2]. In comparison to ablative laser resurfacing, nonablative lasers are less aggressive, keeping the epidermis intact and leading to minimal downtime. Fraxel laser, featuring two lasers with different wavelengths (1550 nm/1927 nm), is a well‐established type of nonablative laser treatment [4]. In a retrospective study reviewing 730 patients with diverse skin types and ethnic backgrounds, Fraxel laser treatment showed significant improvements in both pigmentation and wrinkles from baseline, with an overall low risk of adverse events [5], demonstrating its effects on reducing wrinkles and scars, improving skin texture, and addressing pigmentation abnormalities [6, 7].

While Fraxel laser treatment is generally gentler than ablative fractional laser, it can still result in post‐procedural downtime and short‐term complications. For example, many patients may encounter edema and erythema in the 24 to 48 h following Fraxel laser treatment [4]. Specific complications, such as post‐inflammatory hyperpigmentation (PIH), are more commonly observed among people with skin of‐color [7, 8]. Therefore, it is crucial to provide immediate postoperative care to accelerate the healing process and reduce postoperative complications following the nonablative fractional Fraxel laser (NAFL) treatment in Asian populations.

These postoperative complications may develop as a result of inflammation and oxidative stress induced by thermal damage from the laser treatment [9, 10]. Antioxidants have demonstrated effectiveness in managing inflammation and mitigating postoperative oxidative stress by neutralizing oxygen radicals [11, 12]. A recent study indicated that the immediate application of a topical serum containing vitamin C, vitamin E, and ferulic acid (CE Ferulic serum, CEF) following fractional laser surgery can diminish postoperative erythema and edema within a week compared to a vehicle control [12]. Furthermore, the safety profile of CEF has been confirmed, showing good tolerance with immediate use after ablative fractional resurfacing procedures [13].

Given the evidence from previous studies, there is potential value in investigating the effects of combining CEF with NAFL treatment. This study aimed to investigate the efficacy and patient satisfaction of CEF following NAFL treatment in the Chinese population.

## 2 Materials and Methods

### 2.1 Study Design and Patients

This study is a randomized, investigator‐blinded, split‐face, controlled trial conducted among patients aged 18 to 65 years old with Fitzpatrick skin type II‐IV. Photoaging patients suitable for Fraxel laser treatment were enrolled, as determined by the investigator, exhibiting skin conditions including melasma, fine lines, enlarged pores, and dullness, etc. Patients were excluded if any of the following criteria were met: (1) history of skin pigmentation, such as due to hormonal factors; (2) received steroids, phototoxic drugs, or received any laser treatment over the past 2 months prior to the enrollment; (3) allergic to any components in CE Ferulic or NAFL treatment; and (4) infections or inflammation present in the skin areas to be treated. Informed consent was obtained from all patients. This trial was approved by the Ethics Committee of the Deyi Skin Institution (IRB: DEYI202308160001).

### 2.2 Interventions and Follow‐Up

**FIGURE 1:**
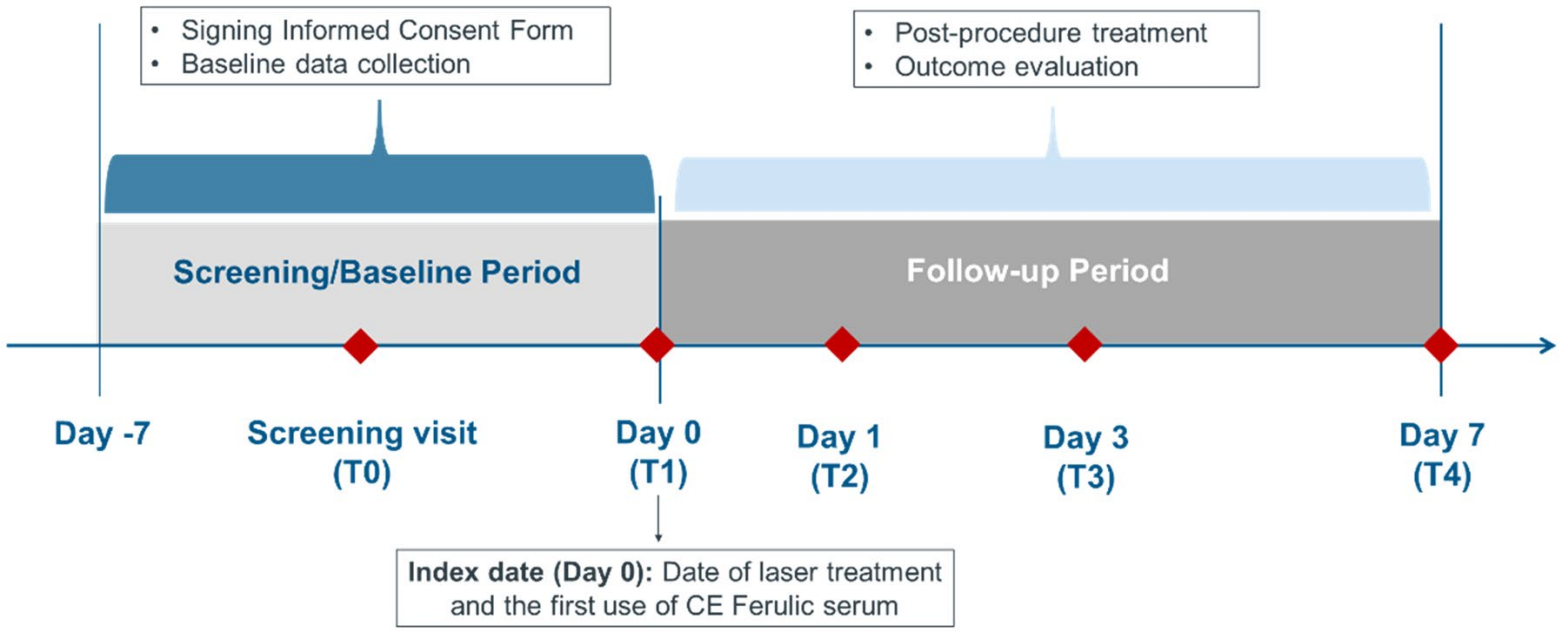
Schematic of study design.

The study consisted of five visits, including the screening visit (T0: Days −7 to 0), the enrollment and treatment visit (T1: Day 0, immediately post‐procedure), and follow‐up visits (T2‐4: post‐procedure Days 1, 3, and 7) (Figure 1).

On the index date (Day 0), all patients received Fraxel laser treatment (device: Fraxel DUAL 1550/1927 Laser System) on both sides of the face. Following the laser treatment, patients were randomly assigned using a random number table to apply CEF (intervention) on either the left or right side of the face in a 1:1 ratio, and to apply 0.9% normal saline (NS; control) on the other side of the face, immediately after the procedure and after their daily morning routine skincare during the 7‐day follow‐up. Sunscreen was also applied once daily. CEF contains 15% vitamin C (L‐ascorbic acid), 1% vitamin E (alpha‐tocopherol), and 0.5% ferulic acid. During the study period, patients were required to record daily videos of their routine skincare process for researchers to check their adherence. Additionally, the investigators would check the consumption of CEF serum and 0.9% normal saline at each follow‐up visit to see if it generally matched the expected consumption.

### 2.3 Endpoints and Assessments

Independent investigators were blinded when assessing the evaluation indicators based on standardized clinical images obtained by the VISIA skin imaging system (Canfield Scientific, USA). The primary endpoint was the change from baseline in erythema score (0 = no erythema, 1 = mild erythema, 2 = moderate erythema, 3 = severe erythema) on Day 7. Key secondary endpoints included the change from baseline in erythema score on Days 1 and 3, the changes from baseline in edema score and skin sebum content, the percentage changes from baseline in erythema index (EI), melanin index (MI), skin hydration, and transepidermal water loss (TEWL), as well as scabbing formation/detachment and patients' overall satisfaction on Days 1, 3, and 7. Clinical evaluation indicators were measured by the multifunctional skin tester MPA20 (Courage Khazaka, Germany) with the Mexameter MX18 probe (for EI and MI), Corneometer CM825 probe (for skin hydration), Tewameter TM300 probe (for TEWL), and Sebumeter SM815 probe (for skin sebum content). Scabbing stage and satisfaction were obtained from a patient questionnaire.

Safety endpoints included patient‐reported pain level using a Visual Analogue Scale (VAS) from score 0 representing “no pain” to score 10 representing “extreme pain” at 15 min, 30 min, and 1 h immediately after the NAFL treatment and on Day 1. Any adverse events (AEs) and serious adverse events (SAEs) starting from the screening process were recorded.

### 2.4 Statistical Analysis

The study required a minimum sample size of 50 patients, assuming an effect size of 1 with a standard deviation (SD) of 0.9 for the primary endpoint, a significance level of 0.05, 80% power, and a 20% dropout rate based on the previous publication [14].

To test the superiority hypothesis for continuous variables, paired t‐tests or Wilcoxon signed‐rank tests were used. For categorical variables, chi‐squared tests were employed, and for ordinal variables, Wilcoxon signed‐rank tests or Cochran's and Mantel–Haenszel statistics were used. A significance level of alpha < 0.05 was considered statistically significant in all analyses. Statistical analyses were conducted using SAS 9.4 and SPSS (version 22.0 or later).

## 3 Results

A total of 50 patients were enrolled in the study from September 4 2023 to October 23 2023. All patients completed the full course of treatment and follow‐up. Overall, the mean ± SD age was 31.6 ± 7.2 years and 90% of the patients were female (45/50). There were no statistically significant differences between the two groups regarding baseline characteristics, except that the erythema index on the NS side was higher than on the CEF side (249.65 ± 57.59 vs. 245.33 ± 58.02, *p* = 0.036) (Table 1).

**TABLE 1:** Skin parameters at baseline.

Parameters	CEF, mean ± SD (*n* = 50)	NS, mean ± SD (*n* = 50)	Difference between groups, mean ± SD	*p*
Erythema score	0.06 ± 0.24	0.06 ± 0.24	0.00 ± 0.00	NA^a^
Erythema index (EI)	245.33 ± 58.02	249.65 ± 57.59	−4.33 ± 23.46	0.036
Melanin index (MI)	145.77 ± 29.63	148.96 ± 30.75	−3.19 ± 14.40	0.124
Skin hydration	71.58 ± 13.13	72.91 ± 11.50	−1.33 ± 7.82	0.235
Transepidermal water loss (TEWL)	10.39 ± 3.51	9.88 ± 3.59	0.51 ± 2.11	0.094
Skin sebum content	10.42 ± 13.39	10.92 ± 14.98	−0.50 ± 8.55	0.911

Abbreviations: CEF, CE Ferulic serum; NA, not applicable; NS, normal saline; SD, standard deviation.

^a^ The *p* value was not applicable because the values of the two groups were completely the same.

### 3.1 Primary Endpoint

**FIGURE 2:**
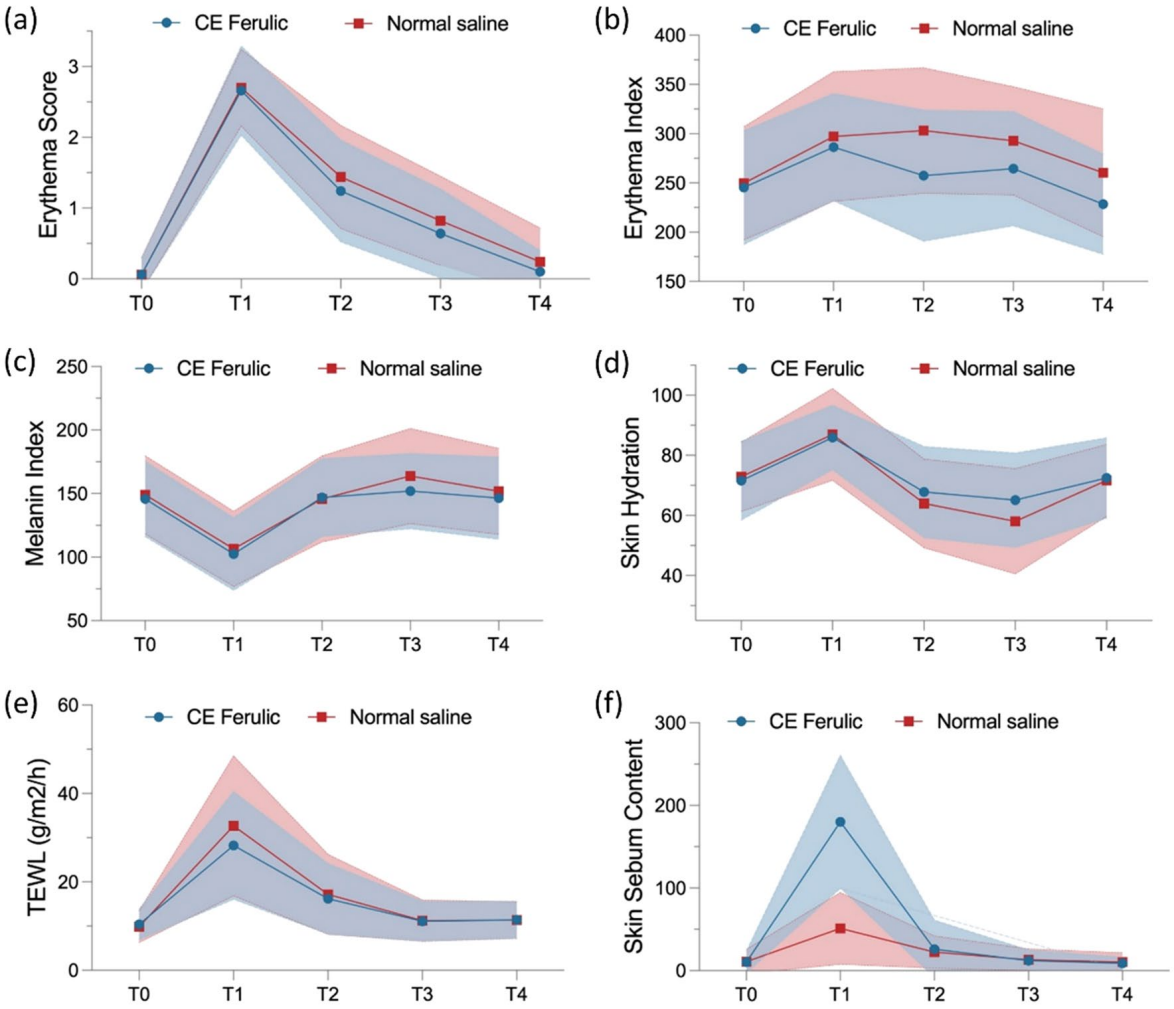
Skin parameters at baseline and during follow‐up (T0: baseline; T1: immediately post‐procedure; T2: Day 1; T3: Day 3; T4: Day 7). (a) Erythema score (b) Erythema index; (c) Melanin index; (d) Skin hydration; (e) Transepidermal water loss (TEWL); (f) Skin sebum content.

A significant difference was observed in the change from baseline in erythema score between the CEF side and the NS side on Day 7 (0.04 ± 0.40 vs. 0.18 ± 0.48, *p* = 0.011) (Table 2). Throughout the 7‐day follow‐up, the erythema score was lower on the CEF side than on the NS side (Figure 2a).

**TABLE 2:** Change in erythema score from baseline on Days 1, 3, and 7.

Erythema score; follow‐up time	CEF, mean ± SD (*n* = 50)	NS, mean ± SD (*n* = 50)	Difference between groups, mean ± SD	*p*
T2‐T0	1.18 ± 0.75	1.38 ± 0.78	−0.20 ± 0.49	0.008
T3‐T0	0.58 ± 0.61	0.76 ± 0.62	−0.18 ± 0.44	0.008
T4‐T0	0.04 ± 0.40	0.18 ± 0.48	−0.14 ± 0.35	0.011

*Note:* T0: baseline; T2: Day 1; T3: Day 3; T4: Day 7; T2–T0: the mean change from baseline on Day 1, T3–T0: the mean change from baseline on Day 3, T4–T0: the mean change from baseline on Day 7.

Abbreviations: CEF, CE Ferulic serum; NS, normal saline; SD, standard deviation.

### 3.2 Secondary Endpoints

#### 3.2.1 EI and MI

In parallel with the erythema score assessed by investigators, the mean percentage change from baseline in EI was lower on the CEF side compared to the NS side during the 7‐day follow‐up (Table 3, Figure 2b). Specifically, on Day 7, the CEF side showed a significant improvement in terms of the mean percentage change from baseline in EI, compared with the NS side (−4.05% ± 22.12% vs. 5.72% ± 20.41%, *p* < 0.001). As for MI, the difference in percentage change from baseline was −7.16% ± 17.15% (*p* = 0.005) between the CEF side and the NS side on Day 3 (Table 3, Figure 2c).

**TABLE 3:** Mean percentage changes from baseline in skin parameters.

Parameters; follow‐up time	Mean percentage changes from baseline ± SD		Difference between groups, mean ± SD	*p*
	CEF (*N* = 50)	NS (*N* = 50)		
Erythema index (EI)				
T2: Day 1	7.57 ± 25.25	23.66 ± 21.37	−16.09 ± 22.66	< 0.001
T3: Day 3	10.50 ± 22.86	20.86 ± 27.25	−10.36 ± 28.61	0.084
T4: Day 7	−4.05 ± 22.12	5.72 ± 20.41	−9.77 ± 17.11	< 0.001
Melanin index (MI)				
T2: Day 1	1.46 ± 13.58	−1.87 ± 13.71	3.33 ± 18.29	0.204
T3: Day 3	3.35 ± 17.46	10.50 ± 17.17	−7.16 ± 17.15	0.005
T4: Day 7	0.95 ± 14.77	2.40 ± 13.30	−1.45 ± 16.38	0.534
Skin hydration				
T2: Day 1	−1.61 ± 32.26	−11.14 ± 21.17	9.53 ± 24.37	0.007
T3: Day 3	−6.64 ± 25.93	−18.44 ± 27.44	11.80 ± 24.94	0.002
T4: Day 7	2.94 ± 19.00	−0.38 ± 17.42	3.32 ± 16.22	0.154
Transepidermal water loss (TEWL)				
T2: Day 1	68.15 ± 91.87	86.97 ± 97.56	−18.83 ± 66.20	0.050
T3: Day 3	19.50 ± 64.07	26.84 ± 59.77	−7.34 ± 56.14	0.360
T4: Day 7	20.34 ± 53.90	26.46 ± 54.45	−6.12 ± 49.37	0.385
Skin sebum content^a^				
T2: Day 1	15.46 ± 39.81	11.46 ± 26.23	4.00 ± 36.72	0.782
T3: Day 3	1.64 ± 16.38	2.22 ± 18.41	−0.58 ± 10.43	0.456
T4: Day 7	−1.58 ± 13.77	−0.62 ± 15.77	−0.96 ± 13.50	0.600

Abbreviations: CEF, CE Ferulic serum; NS, normal saline; SD, standard deviation.

^a^ Skin sebum content was the mean changes from baseline, while the other parameters were the mean percentage changes from baseline; T2: Day 1; T3: Day 3; T4: Day 7.

#### 3.2.2 Skin Hydration, TEWL, and Skin Sebum Content

The differences in the percentage changes in skin hydration from baseline between the CEF side and the NS side were 9.53% ± 24.37% (*p* = 0.007) and 11.80% ± 24.94% (*p* = 0.002) on Day 1 and Day 3, respectively. The CEF side showed a lower percentage increase in TEWL compared to the NS side over the 7‐day follow‐up, although no significant differences were observed. There were no significant differences in the change in skin sebum content between the CEF side and the NS side over follow‐up (Table 3, Figure 2d–f).

#### 3.2.3 Skin Edema and Scabbing Detachment Stage

In terms of edema score, there was a trend of reduction throughout the follow‐up period. No significant difference was observed regarding scabbing detachment stage between the CEF side and the NS side over follow‐up.

#### 3.2.4 Patients' Overall Satisfaction

**FIGURE 3:**
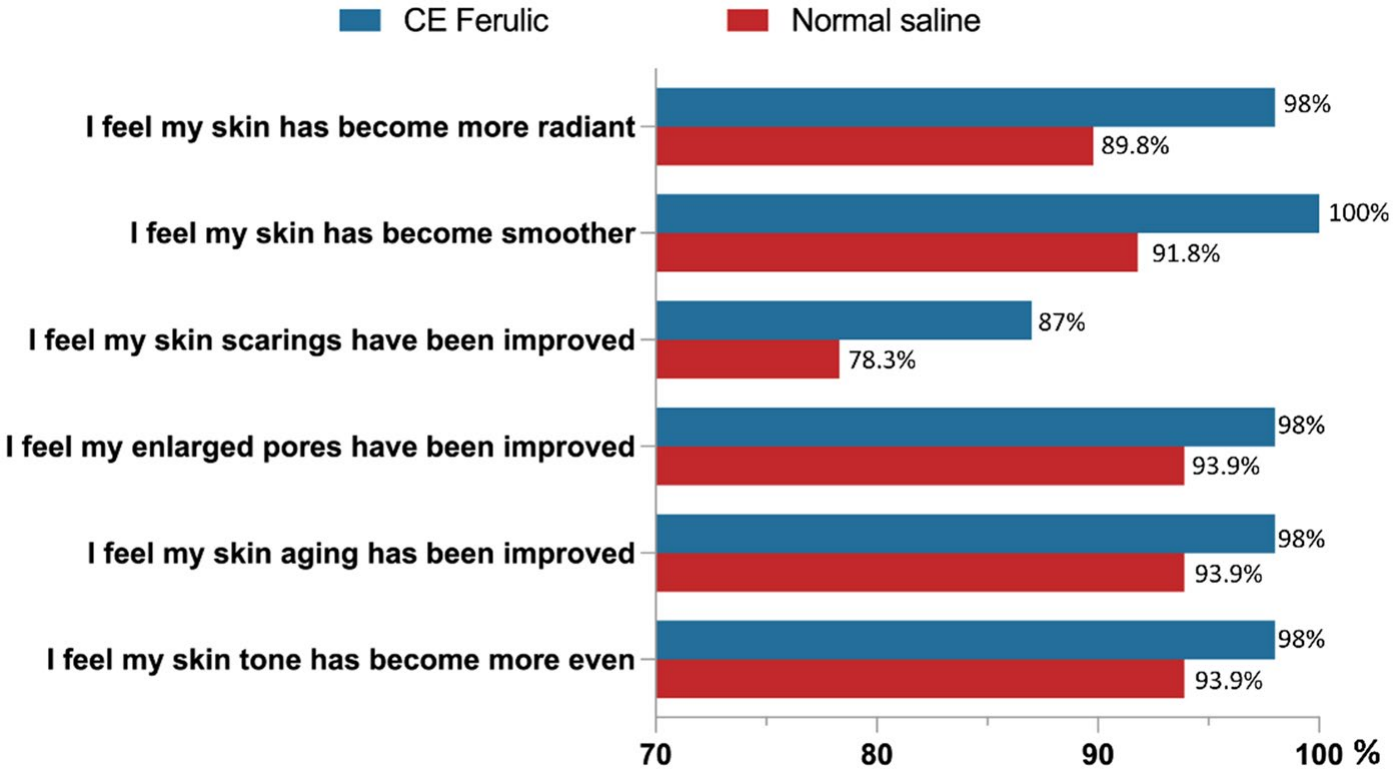
Patients' satisfaction on Day 7 after nonablative fractional Fraxel laser treatment.

On Day 7, patients were more satisfied with the CEF side than the NS side in several attributes of skin quality, including skin unevenness, skin aging, enlarged pores, scarring, skin smoothness, and radiance (Figure 3). More patients were very satisfied with the efficacy (CEF: 59.2% vs. NS: 46.9%) and comfort (CEF: 53.1% vs. NS: 38.8%) of the CEF side than that of the NS side. Furthermore, regarding overall satisfaction, a total of 49 patients completed the questionnaire on Day 7. Among them, 49% (24/49) of the patients thought the outcome of the CEF side exceeded their expectations and no one thought it was below their expectations, while 4.1% (2/49) of the patients thought the outcome of the NS side did not meet their expectations.

**FIGURE 4:**
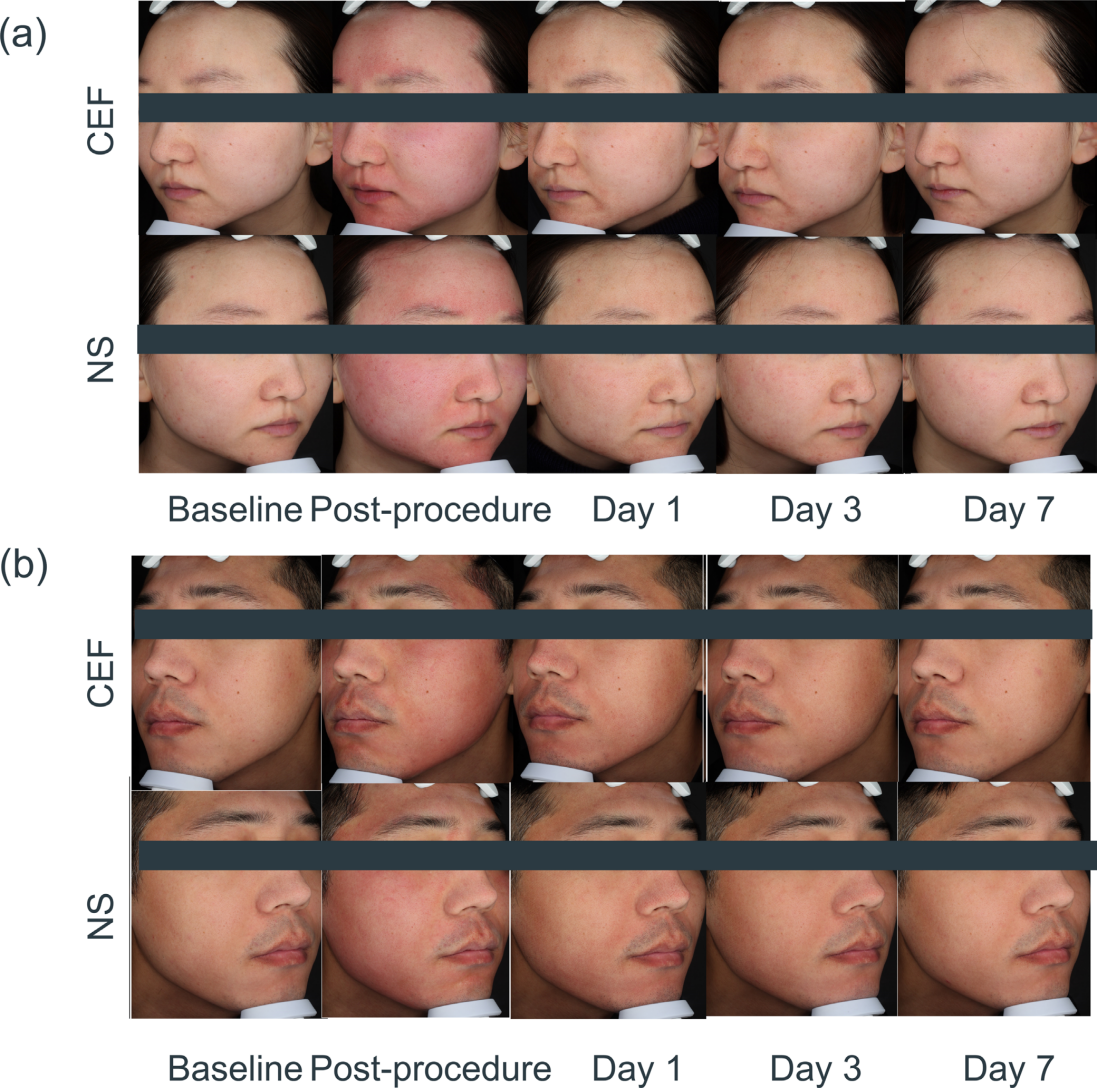
Clinical photographs of facial skin at baseline and during follow‐up. (a) Patient 1: a 27‐year‐old female with enlarged pores; (b) Patient 2: a 36‐year‐old male with acne scars. CEF, CE Ferulic serum; NS, normal saline.

Clinical photographs of both sides of the face in two patients at baseline, post‐procedure, and during the follow‐up period are displayed in Figure 4.

### 3.3 Safety Endpoints

Patients experienced significantly less pain on the CEF side compared with the NS side at both 30 min and 1‐h following NAFL treatment (Table 4). No product‐related adverse events were reported.

**TABLE 4:** Pain score by VAS.

Follow‐up time	CEF, mean ± SD (*n* = 50)	NS, mean ± SD (*n* = 50)	Difference between groups, mean ± SD	*p*
15 min	5.60 ± 1.56	5.76 ± 1.49	−0.16 ± 0.82	0.218
30 min	3.62 ± 1.37	4.02 ± 1.15	−0.40 ± 1.21	0.036
1 h	1.84 ± 1.27	2.22 ± 1.09	−0.38 ± 1.07	0.022

Abbreviations: CEF, CE Ferulic serum; NS, normal saline; SD, standard deviation; VAS, Visual Analogue Scale.

## 4 Discussion

This randomized, investigator‐blinded, split‐face, controlled trial examined the efficacy and patient satisfaction of applying CEF following NAFL treatment among patients with facial photodamage in China. The study showed that combining CEF with Fraxel laser led to improved erythema score, EI, MI, and skin hydration within 1 week following the Fraxel laser treatment, as well as a higher level of patient satisfaction and less pain.

Although NAFL is an effective treatment to address the signs of photoaging, the procedure can still result in an inflammatory reaction and oxidative stress, leading to complications such as erythema and hyperpigmentation. This study demonstrated improvements in erythema score, EI, and MI after treatment with CEF following NAFL treatment than NS, which may be attributed to the antioxidant properties of vitamin C and E. The decreased erythemal reaction on the CEF‐treated side indicates a suppressed inflammatory response, thus potentially leading to a reduced risk of PIH. Additionally, vitamin C is known for its depigmenting effects by interacting with the enzyme tyrosinase and reducing melanin formation [15].

This study also demonstrated that the application of CEF was associated with skin barrier recovery, as evidenced by a significant improvement in skin hydration and a tendency towards reduced TEWL. Consistent with these results, a previous study in the Republic of Korea investigated the effect of vitamin C as a post‐Fraxel laser regimen in 44 female patients and found that vitamin C treatment was associated with improvements in post‐operative TEWL and restoration of skin pH compared to no treatment [16]. Beneficial effects were also reported in terms of improved skin hydration following the topical application of ferulic acid [17, 18].

NAFL is recognized for its gentle approach to skin rejuvenation and resurfacing, which may explain the minimal changes in edema and scabbing formation or detachment after Fraxel treatment in this study. In our study, patients reported experiencing milder pain sensations following the procedure when CEF was applied immediately, resulting in higher comfort levels and overall satisfaction on the CE Ferulic side. This observation could be attributed to the analgesic effect of vitamin C [19]. While the mechanisms remain unclear, vitamin C is believed to reduce pro‐inflammatory mediators that trigger pain and may be involved in neuromodulation as a cofactor in the synthesis of neurotransmitters [19].

In this study, the combined use of CEF post‐procedure has been shown to enhance the benefits of NAFL in treating photoaging while reducing the potential complications. Furthermore, a previous study has indicated a dose‐dependent permeation of CEF in response to increased laser treatment levels, suggesting that the minor skin damage caused by NAFL can enhance the absorption of active components in CE Ferulic, leading to a synergistic effect and optimal treatment outcomes [20]. This integrated skincare regimen provides a comprehensive approach to post‐laser treatment care. Given that Asian skin has more melanocytes and tends to produce more melanin, making this population more susceptible to pigmentation disorders, it is essential to employ this integrated skincare approach to optimize the effectiveness and minimize the complications of NAFL treatment in this population [21].

One limitation of this study is that patients were unblinded for satisfaction and pain assessment, which may influence their judgments. Another limitation is the potential for variation in adherence and application techniques among individuals, despite the various approaches we have implemented to enhance adherence. Nonetheless, this approach mirrors real‐world daily skincare routines, offering a reasonable estimate of the effectiveness of CEF after Fraxel laser treatment. In addition, participants in this study received only one session of Fraxel laser treatment, whereas this treatment usually requires several sessions in clinical practice. Therefore, further research should be conducted to examine the long‐term effectiveness of CEF following the entire laser treatment course.

## 5 Conclusions

This is the first trial to provide clinical evidence among Chinese patients with facial photodamage using CEF serum after NAFL. It demonstrated that applying CEF serum after a Fraxel laser procedure led to improvements in reducing erythema, maintaining skin hydration, and promoting the healing process more than NS. The results indicate a beneficial effect of the combination of CE Ferulic with Fraxel laser treatment.

## Author Contributions

The authors confirm contribution to the paper as follows: study conception and project administration: Kai Li; Investigation: Xiaolei Qin; data collection: Chenxi Zhou, Yanmei Wang, Mengjiao Chen and Liyuan Zhang, Xiao Luo, Lin Zhu, Qi Shi and Weiliang Chen; analysis and interpretation of results: Jinlong Zhai; All authors reviewed the results and approved the final version of the manuscript.

## Ethics Statement

This trial was approved by the Ethics Committee of the Deyi Skin Institution (IRB: DEYI202308160001).

## Consent

We confirm that informed consent has been obtained from all identifiable participants featured in the photographs included in this article. Each participant has provided consent via a signed consent form, in accordance with the guidelines. The required consent forms are available in appendix.

## Conflicts of Interest

L'Oréal provided funding for the trial. Study materials (CE Ferulic serum) were provided by SkinCeuticals Inc. There were no other grants or fundings to be disclosed by Xiaolei Qin, Jinlong Zhai, Chenxi Zhou, Yanmei Wang, Mengjiao Chen, Lin Zhu, Qi Shi, Weiliang Chen, Liyuan Zhang, Xiao Luo, and Kai Li.

## Data Availability

The data that support the findings of this study are available on request from the corresponding author. The data are not publicly available due to privacy or ethical restrictions.
